# Effects and mechanism of renal denervation on ventricular arrhythmia after acute myocardial infarction in rats

**DOI:** 10.1186/s12872-022-02980-4

**Published:** 2022-12-12

**Authors:** Jian Ye, Rongxue Xiao, Xu Wang, Ruiqing He, Zongjun Liu, Junqing Gao

**Affiliations:** 1 Shanghai Putuo Central School of Clinical Medicine, Anhui Medical University, Shanghai, 200062 People’s Republic of China; 2 The Fifth School of Clinical Medicine, Anhui Medical University, Shanghai, 200062 People’s Republic of China; 3 Department of Cardiology, Putuo Hospital, Shanghai University of Traditional Chinese Medicine, Shanghai, 200062 People’s Republic of China

**Keywords:** Renal denervation, Acute myocardial infarction, Ventricular arrhythmia, Central nervous system, Sympathetic nerve

## Abstract

**Background:**

Renal denervation (RDN) can reduce ventricular arrhythmia after acute myocardial infarction (AMI), but the mechanism is not clear. The purpose of this study is to study its mechanism.

**Methods:**

Thirty-two Sprague–Dawley rats were divided into four groups: control group, AMI group, RDN-1d + AMI group, RDN-2w + AMI group. The AMI model was established 1 day after RDN in the RDN-1d + AMI group and 2 weeks after RDN in the RDN-2w + AMI group. At the same time, 8 normal rats were subjected to AMI modelling (the AMI group). The control group consisted of 8 rats without RDN intervention or AMI modelling.

**Results:**

The study confirmed that RDN can reduce the occurrence of ventricular tachycardia in AMI rats, reduce renal sympathetic nerve discharge, and inhibit the activity of local sympathetic nerves and cell growth factor (NGF) protein expression in the heart after AMI. In addition, RDN decreased the expression of norepinephrine (NE) and glutamate in the hypothalamus,and NE in cerebrospinal fluid, and increased the expression level of γ aminobutyric acid (GABA) in the hypothalamus after AMI.

**Conclusion:**

RDN can effectively reduce the occurrence of ventricular arrhythmia after AMI, and its main mechanism may be via the inhibition of central sympathetic nerve discharge.

**Supplementary Information:**

The online version contains supplementary material available at 10.1186/s12872-022-02980-4.

## Background

Acute myocardial infarction (AMI) refers to the acute and persistent insufficiency of coronary blood supply leading to myocardial ischaemic necrosis. The incidence of AMI is increasing every year, the affected population tends to be younger, and AMI has become one of the major diseases that cause death and disability in humans. Ventricular arrhythmia caused by AMI is the main factor leading to sudden cardiac death and an indicator of poor prognosis of patients [1]. Cardiac sympathetic nerve remodelling and abnormal excitation after AMI are important causes of ventricular arrhythmia [2]. Renal denervation (RDN) is an interventional procedure that has emerged in recent years. It can inhibit the activation of the renin angiotensin aldosterone system (RAAS) and decrease systemic sympathetic nerve activity. Initially, RDN was mainly used to treat hypertension, but now more and more evidence shows that RDN is beneficial also to other entities including heart failure, arrhythmia, cardiac hypertrophy [3–6]. Studies have shown that RDN can inhibit the occurrence of ventricular arrhythmia during ischemia/AMI [7]. At the same time, a number of clinical studies have confirmed that RDN can effectively reduce various types of ventricular arrhythmias [8–13], but its mechanism of action is not clear. Aim of this study was to explore the effect of RDN on ventricular arrhythmias during ischemia/AMI and the underlying mechanism.

## Methods

### Animals

A total of 32 SD male rats, weighing 200–250 g, were purchased from Shanghai Slack Laboratory Animal Co., Ltd. (Certificate Number: 20170005043887) and were raised under standard conditions. All experimental procedures were approved by the Shanghai Medical Ethics Committee. The 32 SD rats were randomly divided into 4 groups by the random number method: the control group, AMI group, RDN-1d + AMI group, and RDN-2w + AMI group. There were 8 rats in each group, and models were established for the corresponding treatment.

### RDN operation

Rats were fasted with access to water before the operation, weighed before anaesthesia, and then anaesthetized with intraperitoneal administration of 2.5% sodium pentobarbital solution (0.3 ml/100 g). After anaesthesia induction, the rats were placed flat and fixed in a supine position on the operating table with a rubber band. The abdominal skin was shaved with a razor and disinfected with a cotton ball soaked in 75% alcohol. A surgical incision was made on the abdominal centreline, sterile gauze soaked in saline was used to cover the intestines, and the bilateral renal artery was isolated. Then, sterile gauze strips soaked in 10% phenol solution with 95% ethanol were gently wrapped around both renal arteries for one week to keep the phenol from contacting the surrounding organs as much as possible, and the gauze covering was removed one minute later [14]. After the operation, the incisions were sutured layer by layer, and penicillin was injected for 3 consecutive days to prevent infection.

### AMI model

SD rats were anaesthetized with 2.5% sodium pentobarbital solution (0.3 ml/100 g) by intraperitoneal injection, fixed, intubated and connected to a ventilator after anaesthesia. Electrodes were connected to the rat, and a PowerLab multichannel physiological recorder was used to collect signals. ECG acquisition software was used to set the measurement parameters. The chest was opened at the third intercostal space on the left side of the sternum to expose the left anterior descending coronary artery. Ligation was performed at the junction point of the distal 1/3 [14], and ECG monitoring showed that the ST segment remained elevated, indicating that establishment of the myocardial infarction model was successful. Then, the signals were recorded continuously for 1 h, and finally, the number of ventricular tachycardia events were counted with the software.

### Histopathological examination

Fresh cardiac and renal artery tissue samples were removed and cleaned. Half of the samples were frozen at −80 °C, and the remaining samples were fixed with 4% paraformaldehyde solution and embedded in paraffin. Haematoxylin and eosin (HE) and tyrosine hydroxylase (TH) staining were used to evaluate the effectiveness of RDN in the renal artery and surrounding sympathetic nerves. Cardiac sympathetic nerve activity was detected by TH and nerve growth factor (NGF) staining.

### Western blot

The myocardial tissue frozen at −80 °C was thawed, and the expression level of NGF in the infarcted area was detected by Western blotting.

### Enzyme-linked immunosorbent assay (ELISA)

The hypothalamus and cerebrospinal fluid were extracted, and the levels of GABA, NE and glutamate in the hypothalamic tissue were quantified with ELISA; moreover, the level of NE in the cerebrospinal fluid was measured with ELISA.

### Renal sympathetic nerve activity (RSNA)

After RDN, the rats were anaesthetized by intraperitoneal injection of 2.5% sodium pentobarbital solution (0.3 ml/100 g) and fixed, and the skin was prepared after anaesthesia. The back skin was cut with scissors, the subcutaneous tissue was cut, and the intersection of the abdominal aorta and the renal artery was fully exposed. Then, the left renal sympathetic nerve was located under a dissecting microscope and dissociated, and the free renal sympathetic nerve was gently placed on a bipolar silver wire recording electrode. The recorded discharge signal was amplified 1000 times through the preamplifier and recorded by the oscilloscope and the Power Lab/8SP instrument of the biological information sampling system, and the discharge waveform displayed in the oscilloscope was observed and determined to be renal sympathetic. After nerve discharge, the renal sympathetic nerve and the electrode were wrapped in silica gel for insulation. After the discharge was stable, the RSNA at the basal level was recorded. Then, the rats were euthanized by injection with an excessive amount of pentobarbital (800 mg/kg) to induce the maximum RSNA (usually occurring within 2–5 min after an overdose of anaesthesia). After the renal sympathetic nerve discharge activity stopped, the RSNA background noise value was recorded (generally occurring within 20–30 min after an overdose of anaesthesia). Finally, the RSNA was calculated and analysed according to the following formula: (basic RSNA-RSNA background noise value)/(maximum RSNA–RSNA background noise value) [15].

### Statistical analysis

The data of each group are expressed as the mean ± standard deviation ($\overline{x }$± s), and the comparison between multiple sample means was performed by one-way analysis of variance. For data with a normal distribution, the pairwise comparison of means was performed with a *t* test. If the variances between the two groups were homogeneous, the LSD test was used; if not, the Games-Howell test was used. Data processing was performed by SPSS 24.0 software, and the difference was considered to be statistically significant when *P* < 0.05.

## Results

### The effect of RDN on the occurrence of ventricular arrhythmia during ischemia/AMI

**Fig. 1:**
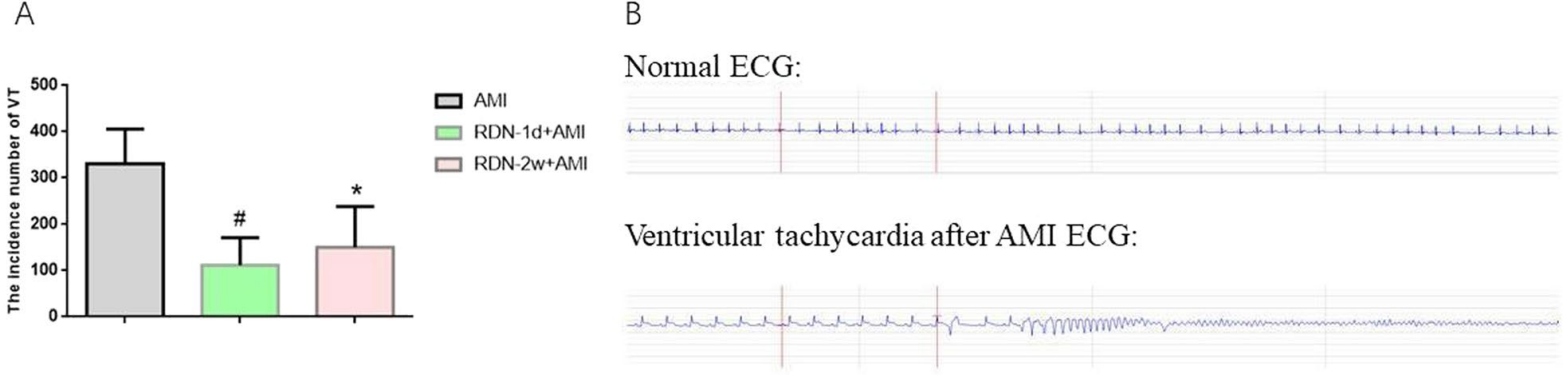
A shows the occurrence frequency of ventricular tachycardia (VT), #RDN-1d + AMI group compared with AMI group, the occurrence frequency of VT decreased, *P*<0.001; *RDN-2w+AMI group compared with AMI group, VT The number of rapid occurrences decreased, *P*=0.003, and the difference was statistically significant. B shows the normal ECG of rats and the ECG manifestations of ventricular tachycardia and ventricular fibrillation after AMI

Verification of the establishment of the AMI model was performed with a PowerLab multichannel recorder, and the output was observed for 1 h. The results showed that compared with the AMI group (331.2 ± 74.8), the RDN-1d + AMI group (112 ± 59.2, *P* < 0.001) and the RDN-2w + AMI group (151 ± 87.9, *P* = 0.003) exhibited a significantly reduction in the occurrence of ventricular tachycardia. Among the groups, the RDN-1d + AMI group showed a more significant decrease in frequency than the RDN-2w-AMI group (Fig. 1). One rat in the AMI group died of ventricular fibrillation, although none of the rats in either RDN group died of ventricular fibrillation.

### HE and TH staining were used to evaluate the damaging effect of RDN on renal sympathetic nerves.

**Fig. 2:**
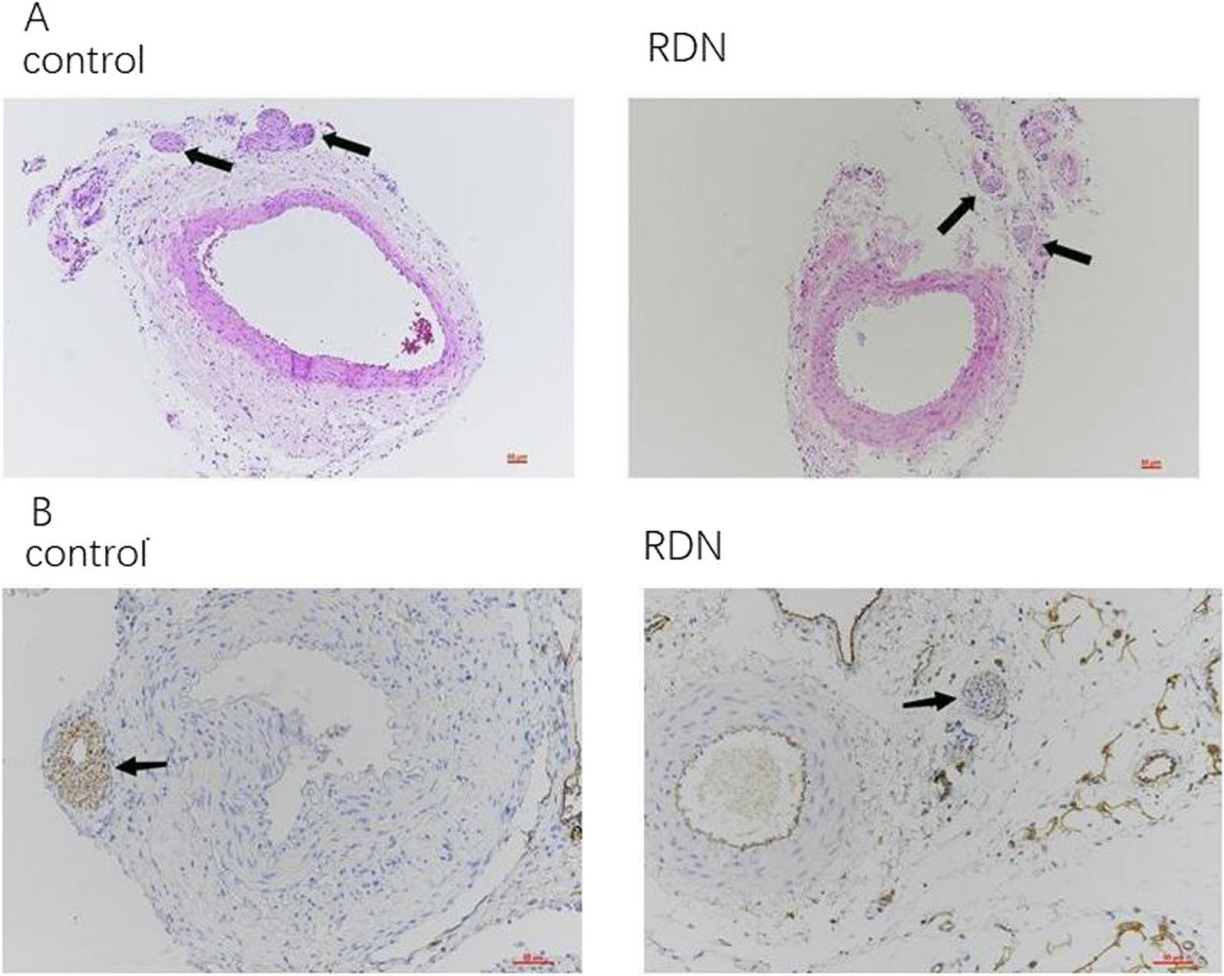
A shows the HE staining of sympathetic nerves around the renal artery of rats (HE × 200). Compared with the Control group, the structure of sympathetic nerve fibers around the renal artery in RDN group changed, the density of sympathetic ganglia was uneven, atrophied, and the number decreased. B shows the TH staining of the sympathetic nerves around the renal artery in rats (TH×200). Compared with the Control group, the sympathetic nerve activity around the renal artery in the RDN group decreased

The formalin-fixed renal artery and surrounding sympathetic nerves were dehydrated, cleared, embedded in wax for HE staining, photographed and analysed under a microscope (Fig. 2A). Compared with the control group, the RDN group showed changes in the tissue structure of sympathetic nerve fibres around the renal artery, unevenly distributed and atrophied sympathetic ganglia, and fewer sympathetic ganglia. Tyrosine hydroxylase (TH) is a key enzyme in the synthesis of catecholamines and is distributed in the cytoplasm of adrenergic axons. Its positive expression can represent the activity of sympathetic nerves in local tissues. The effect of RDN on renal sympathetic nerves was observed using TH staining (Fig. 2B), and the results showed that the phenol chemical ablation method can damage the sympathetic nerves around the renal artery.

### TH staining and NGF staining were used to evaluate cardiac sympathetic activity

**Fig. 3:**
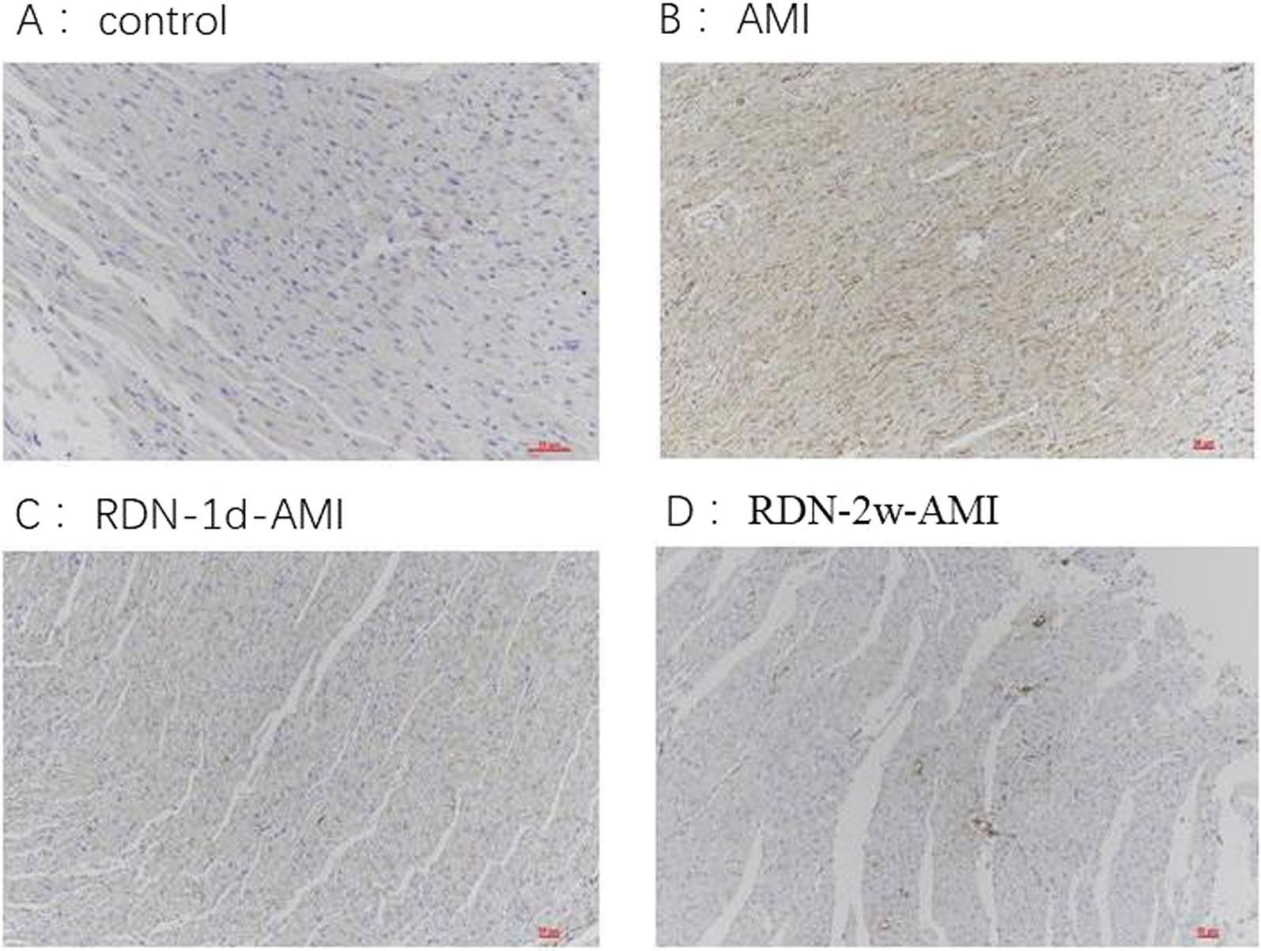
Shows the TH staining of the rat hearts in each group (TH × 200). Compared with the AMI group, the expression of TH in the RDN group was significantly reduced

**Fig. 4:**
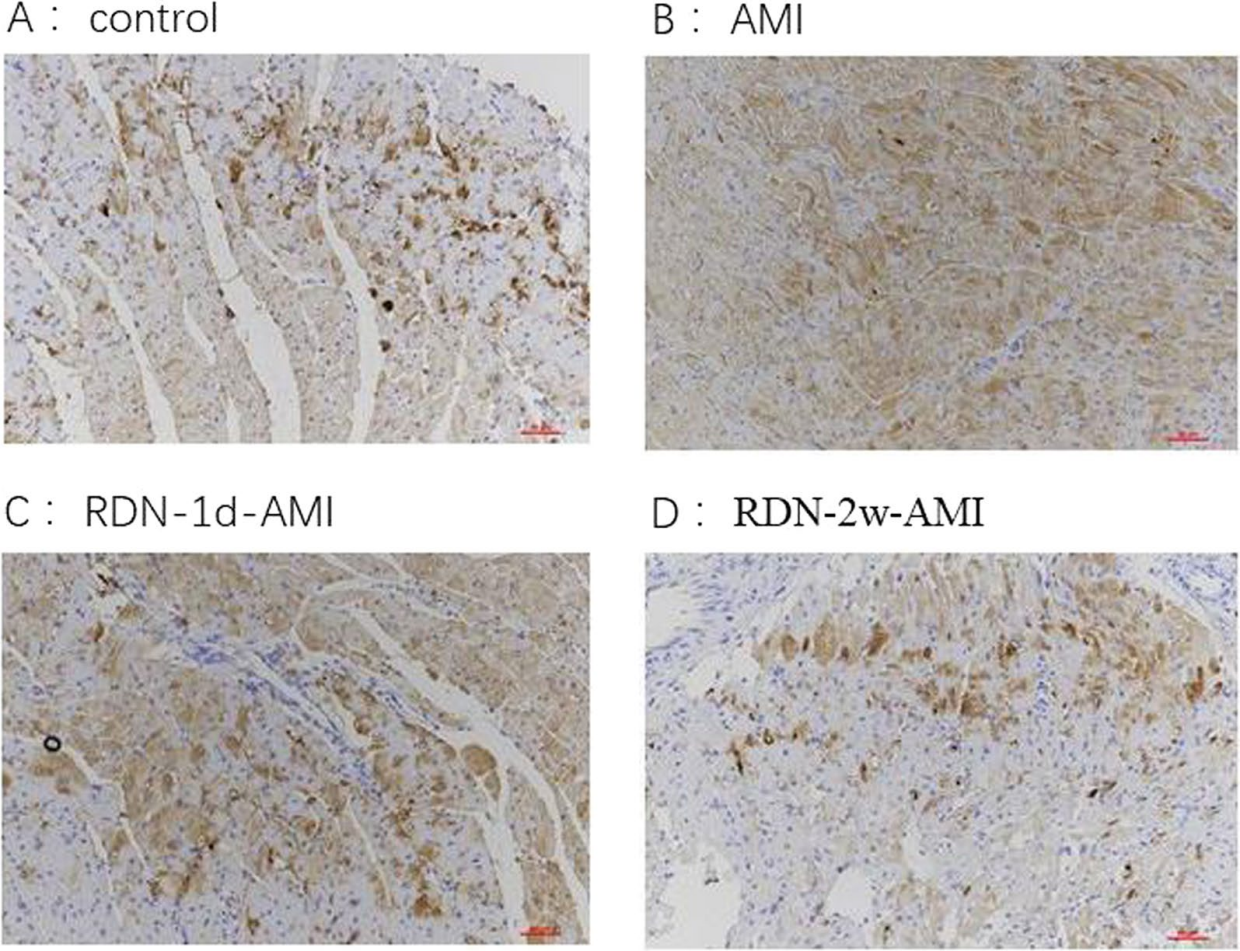
Shows the myocardial NGF staining of rats in each group. Compared with the control group, the expression of NGF increased after AMI and the distribution was uneven; compared with the AMI group, the expression of NGF decreased after RDN, and the decrease in the RDN-2w+AMI group was more obvious

TH staining of paraffin-embedded myocardial tissue sections revealed that the expression of TH in myocardial tissue decreased significantly in the RDN groups compared with the AMI group (Fig. 3). NGF regulates the growth and development of peripheral and central neurons and maintains neuronal survival. Staining for NGF can reflect the functional state of local tissue nerves. Compared with that in the AMI group, the expression of NGF in myocardial tissue decreased after RDN (Fig. 4).

### Western blotting was used to detect NGF expression in the infarcted myocardium

**Fig. 5:**
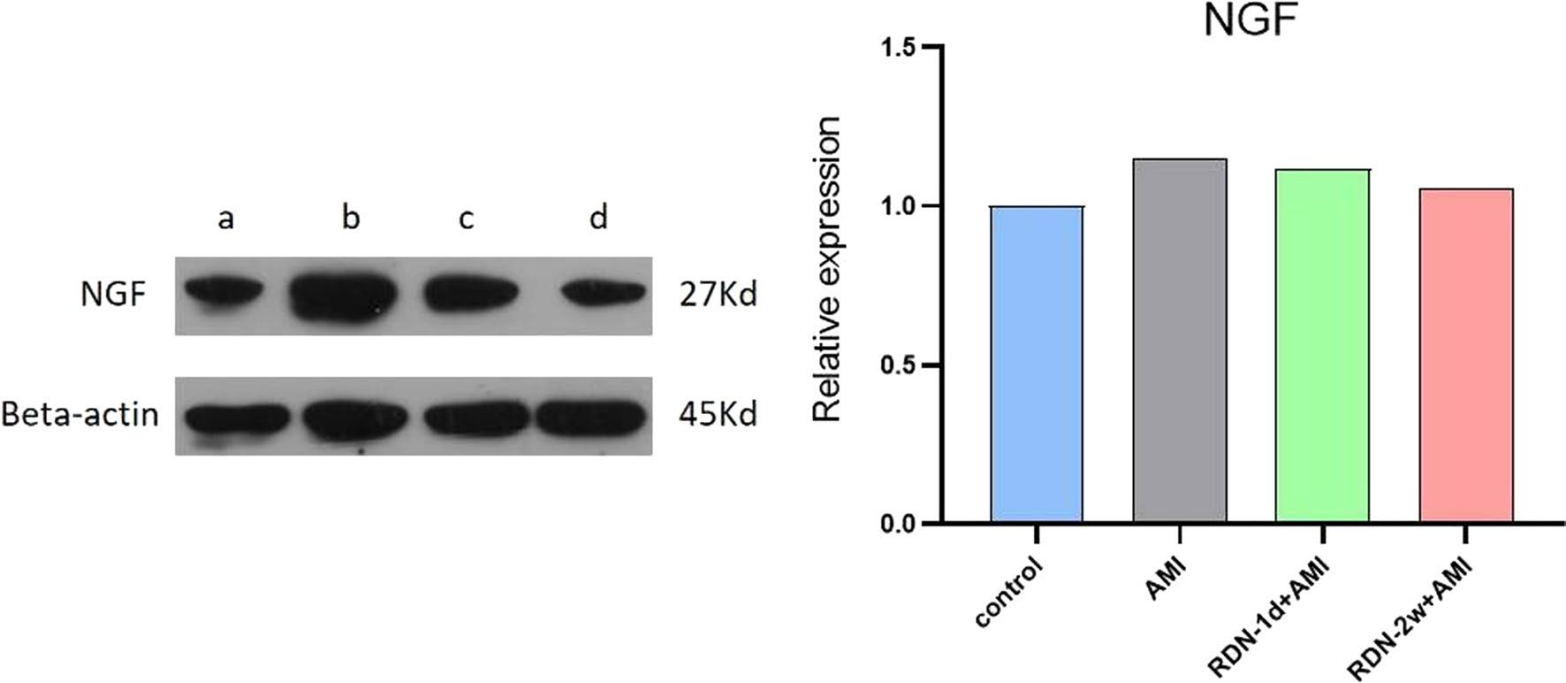
Shows blot image and relative expression level of NGF, **a**–**d** Represent the control group, the AMI group, the RDN-1d + AMI group, and the RDN-2w + AMI group, respectively.(Original blot image，see additional file 1-4.)

NGF protein expression was upregulated in the AMI group compared with the control group and downregulated in the RDN-2w + AMI group compared with the AMI group. However, NGF protein expression was not downregulated in the RDN-1d + AMI group (Fig. 5).

### Detection of renal sympathetic nerve activity

**Fig. 6:**
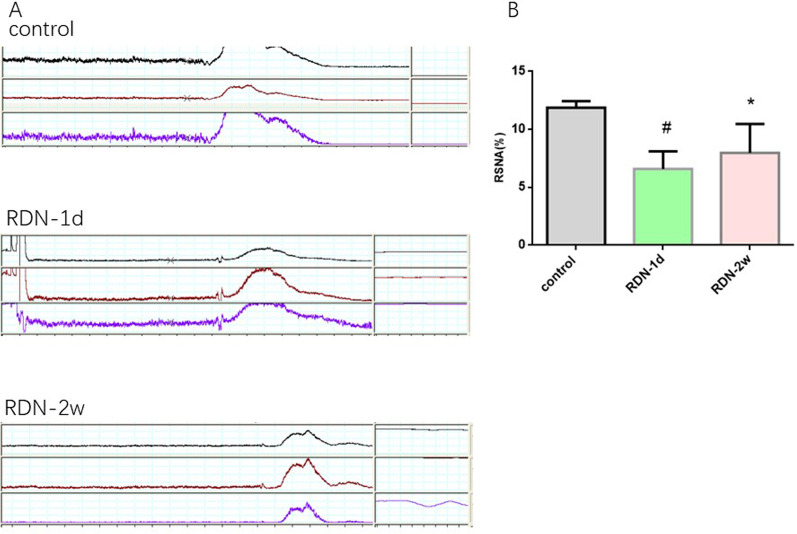
A is the sample of renal sympathetic nerve discharge in each group, B is the activity of renal sympathetic nerve discharge, #Compared with the control group, the renal sympathetic nerve discharge in the RDN-1d group was significantly reduced (*P*<0.001);*Compared with the control group, the renal sympathetic nerve discharge in the RDN-2w group was also significantly decreased (*P*=0.033)

The renal sympathetic nerve discharge signal was recorded by a Power Lab/8SP instrument. Compared with that in the control group (11.9 ± 0.5), the renal sympathetic nerve discharge in the RDN-1d + AMI group was significantly decreased (6.6 ± 1.5, *P* < 0.001). The renal sympathetic nerve discharge was also significantly reduced in the RDN-2w + AMI group (8.0 ± 2.5, *P* = 0.033), and the discharge reduction was more obvious in the RDN-1d + AMI group (Fig. 6).

### The effect of RDN on NE levels in cerebrospinal fluid

Compared with that of the control group, the level of NE in the cerebrospinal fluid of the AMI group was significantly increased (*P* < 0.001). Moreover, compared with that of the AMI group, the level of NE in the cerebrospinal fluid of the RDN-1d + AMI group was significantly decreased (*P* < 0.001), while the level of NE in the cerebrospinal fluid of the RDN-2w + AMI group was significantly lower than that of the RDN-1d + AMI group (*P* < 0.001) (Table 1).

**Table 1:** Comparison of NE content in cerebrospinal fluid of each group ($\overline{x }$±s)

Group	NE (pg/ml)
Control	1040.88 ± 278.76
AMI	3206.75 ± 556.75^*^
RDN-1d + AMI	2103.13 ± 245.39^#^
RDN-2w + AMI	1848.88 ± 351.93^#^
*P*	< 0.001

Compared with the control group,*P < 0.001; compared with the AMI group, ^#^*P* < 0.001

### The effect of RDN on the levels of NE, GABA and glutamate in the hypothalamus

Compared with those of the control group, the levels of NE and glutamate in the hypothalamus of the AMI group was significantly increased (*P* < 0.001), while the level of GABA was decreased (*P* < 0.001). Compared with those of the AMI group, NE and glutamate levels in the hypothalamus of the RDN-1d + AMI group and RDN-2w + AMI group decreased (*P* < 0.001), and the GABA level increased (*P* < 0.001). There was no significant difference in the levels of the three neurotransmitters in the hypothalamus between the RDN-1d + AMI group and the RDN-2w + AMI group (*P* > 0.05) (Table 2).

**Table 2:** Comparison of the contents of NE, GABA and glutamate in the hypothalamus of each group ($\overline{x}$ ± s)

Group	GABA (ng/mg)	NE (pg/mg)	Glutamate (pg/mg)
Control	250.63 ± 26.26	305.50 ± 35.43	303.38 ± 38.21
AMI	120.50 ± 12.32^*^	408.75 ± 58.30^*^	495.88 ± 73.26^*^
RDN-1d + AMI	219.88 ± 18.63^#^	343.13 ± 37.26^#^	356.63 ± 45.65^#^
RDN-2w + AMI	216.00 ± 20.65^#,##^	351.19 ± 54.09^#,##^	358.50 ± 36.12^#,##^
*P*	< 0.001	< 0.001	< 0.001

Compared with control group, *P < 0.001; Compared with AMI group, ^#^*P* < 0.001; Compared with RDN- 1D + AMI group, ^##^*P* > 0.05

## Discussion

**Fig. 7:**
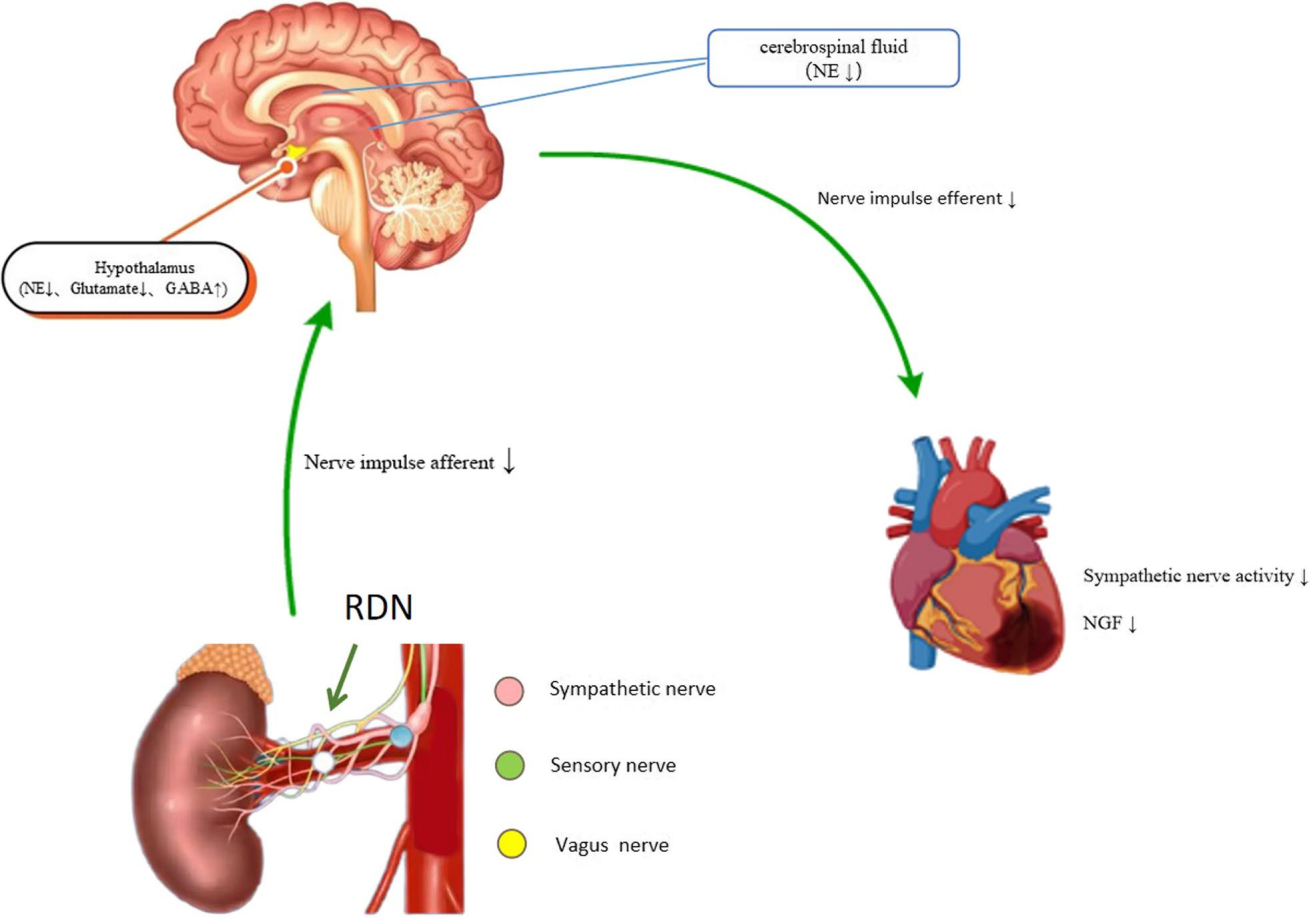
Central illustration figure

The mechanism of ventricular arrhythmia caused by AMI is not clear, but it is generally believed that the sympathetic nerve plays a key role in arrhythmia [16] and that inhibiting sympathetic nerve activity may reduce arrhythmia during ischemia/AMI [17]. AMI leads to nerve damage. Then, the sympathetic nerve sprouts to the ischaemic area, and ischaemic stimulation at the edge of the infarct leads to local hyperinnervation of the myocardium. The combined effect of enhanced sympathetic nerve germination and electrical remodelling of the myocardium is a possible cause of ventricular tachycardia, ventricular fibrillation and sudden cardiac death [18]. RDN damages renal sympathetic nerves, reduces sympathetic nerve activity and inhibits the RAAS system; it is widely used to treat clinical cardiovascular diseases, including hypertension and heart failure [19, 20]. Previous studies have found that RDN can inhibit myocardial remodelling and cardiac electrical remodelling after AMI [21] and can reduce arrhythmias during myocardial ischaemia [7]. In this study, the rats were pretreated with RDN, and then the AMI model was established to compare the occurrence of ventricular arrhythmia during ischemia/AMI. The current study showed that the frequency of ventricular tachycardia in the RDN groups was significantly reduced, and the effect was more pronounced in the RDN-1d + AMI group. During the experiment, one rat in the AMI group died of ventricular fibrillation, although none of the rats in the RDN groups died of ventricular fibrillation, showing that RDN can inhibit the occurrence of ventricular arrhythmia after AMI, thereby reducing mortality.Moreover, our study shows that RDN may reduce the afferent of renal sympathetic nerve impulses, inhibit central sympathetic nerve activity and cardiac sympathetic activity caused by AMI,thereby reducing ischaemic AMI arrhythmias(Fig. 7).

Nerve sprouting and sympathetic excitation after myocardial infarction play an important role in the occurrence of arrhythmia. Studies have reported that inhibiting nerve remodelling after AMI can effectively reduce the occurrence of arrhythmia in rats [22, 23]. Partial myocardial denervation by interventional surgery can effectively prevent AMI-induced ventricular arrhythmias [2]. Enhanced sympathetic nervous system activity of secreted NE is known to bind to β receptors and thus promote the expression of Th and NGF [24].Our study found that AMI can lead to an increase in the expression of TH and NGF in myocardial tissue, which is similar to previous studies [25, 26],indicating that the activity of the cardiac local sympathetic nerve in the AMI group was enhanced. The expression of TH and NGF in myocardial tissue after RDN was lower than after AMI without RDN, especially in the RDN-2w + AMI group. This finding indicates that RDN can inhibit the abnormal excitation of cardiac sympathetic nerves caused by AMI, which is similar to the results of previous studies [27–30]. Compared with RDN-1d + AMI group, NGF decreased more significantly in RDN-2 W + AMI group, suggesting that RDN may not change the expression of Th and NGF immediately, with a hysteresis. Wanying Jiang et al. [31] found that RDN significantly reduced the expression of Th and connexin 43 in the infarct zone of AMI rats compared with metoprolol, which suggests that RDN may not only decrease myocardial sympathetic activity in AMI via NE/β receptor signaling pathway, but may also be associated with the inhibition of inflammation, the RASS system [27, 32].However, the mechanism by which RDN weakens the sympathetic activity of myocardial tissue is not clear.

The central nervous system can be divided into high-level and low-level centres; the low-level centre is located in the spinal cord, and the high-level centre is located in the hypothalamus, the brainstem and the reticular formation. The paraventricular nucleus (PVN) of the hypothalamus plays a major role in central cardiovascular and volume control and regulates sympathetic nerves [33]. Activating the sympathetic nerve by stimulating the PVN can reduce the threshold and increase the occurrence of ventricular fibrillation [34]. Glutamate can act on the PVN to cause sympathetic excitation, and its effect can be inhibited by GABA [35]. Interestingly, the inhibition of PVN activity can reduce arrhythmia in AMI rats [36]. Our study found that after AMI, the level of NE in the hypothalamus and cerebrospinal fluid increased; moreover, the level of the excitatory neurotransmitter glutamate also increased in the hypothalamus, while the level of the inhibitory neurotransmitter GABA decreased in the hypothalamus. RDN can inhibit the increase in NE and glutamate levels caused by AMI and increase the level of GABA in the hypothalamus, which has been reported in previous studies [37] and once again indicates that RDN can reduce central sympathetic nerve activity.

In this study, the discharge of renal sympathetic nerve was significantly reduced after RDN, which means that after RDN, the nerve impulse transmitted from the renal sympathetic nerve to the central nervous system was reduced. RDN damages renal sympathetic nerve, resulting in reduced discharge of it [38], compared with RDN-2 W + AMI group, the discharge of RDN-1d + AMI group decreased more significantly, which may be caused by the self-compensation of preserved nerves.Hong Zheng et al. [39] found that the outflow of the lumbar sympathetic nerve decreased after RDN. This finding, combined with the results of the current study, shows that RDN may reduce central sympathetic nerve activity by reducing the activity of the peripheral sympathetic nerve via a reduction in afferent renal sympathetic nerve activity. This is also a possible mechanism of the relative weakening of cardiac sympathetic activity and the reduction in the occurrence of ventricular arrhythmia in RDN rats after AMI. Interestingly, compared with the RDN-2w + AMI group, the RDN-1d + AMI group showed fewer arrhythmias, lower levels of NE or glutamate in the hypothalamus and cerebrospinal fluid, while the cardiac sympathetic activity in the RDN-2w + AMI group was lower than that in the RDN-1d + AMI group, which suggests that ischemia/AMI-arrhythmias may not only be dependent on central sympathetic excitation.

## Conclusion

The above results suggest that the possible mechanism by which RDN inhibits ventricular arrhythmia caused by AMI is a reduction in afferent renal sympathetic nerve impulses and inhibition of the central sympathetic nerve activity caused by AMI. Then, the central nervous system reduces sympathetic output, thereby reducing cardiac sympathetic activity and the occurrence of ventricular arrhythmia caused by AMI.

## Supplementary Information

**Additional file 1**. (Cuted blot images).

**Additional file 2**. (Original blot images).

**Additional file 3**. (Original blot images).

**Additional file 4**. (Original blot images).

## Data Availability

The datasets used and analyzed during the current study are available from the corresponding author on reasonable request.

## Abbreviations

AMI: Acute myocardial infarction
ECG: Electrocardiogram
ELISA: Enzyme-linked immunosorbent assay
GABA: γ-Aminobutyric acid
NE: Norepinephrine
NGF: Nerve growth factor
PVN: Paraventricular nucleus
RDN: Renal denervation
RAAS: Renin angiotensin aldosterone system
RSNA: Renal sympathetic nerve activity
TH: Tyrosine-hydroxylase
