# Supplementary material for: Effects and mechanism of renal denervation on ventricular arrhythmia after acute myocardial infarction in rats
Source: BMC Cardiovasc Disord. 2022 Dec 12;22:544. doi: 10.1186/s12872-022-02980-4 (PMC9743565; doi:10.1186/s12872-022-02980-4)
Supplement: Supplementary file 1 — Additional file 1. (Cuted blot images). [file 12872_2022_2980_MOESM1_ESM.pptx]

## Slide 1
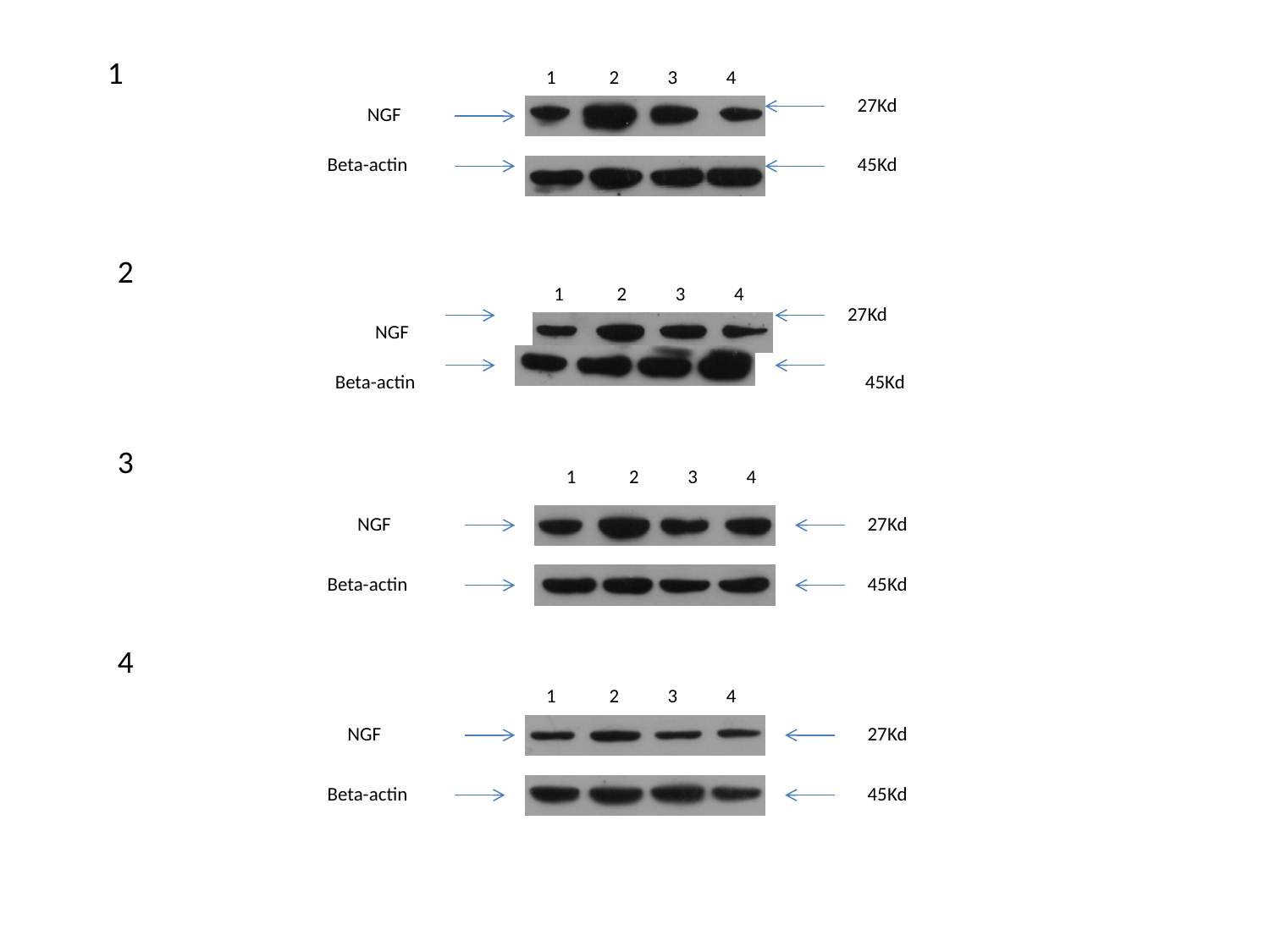

1
 1 2 3 4
27Kd
NGF
Beta-actin
45Kd
2
 1 2 3 4
27Kd
NGF
Beta-actin
45Kd
3
 1 2 3 4
NGF
27Kd
Beta-actin
45Kd
4
 1 2 3 4
NGF
27Kd
Beta-actin
45Kd

## Slide 2
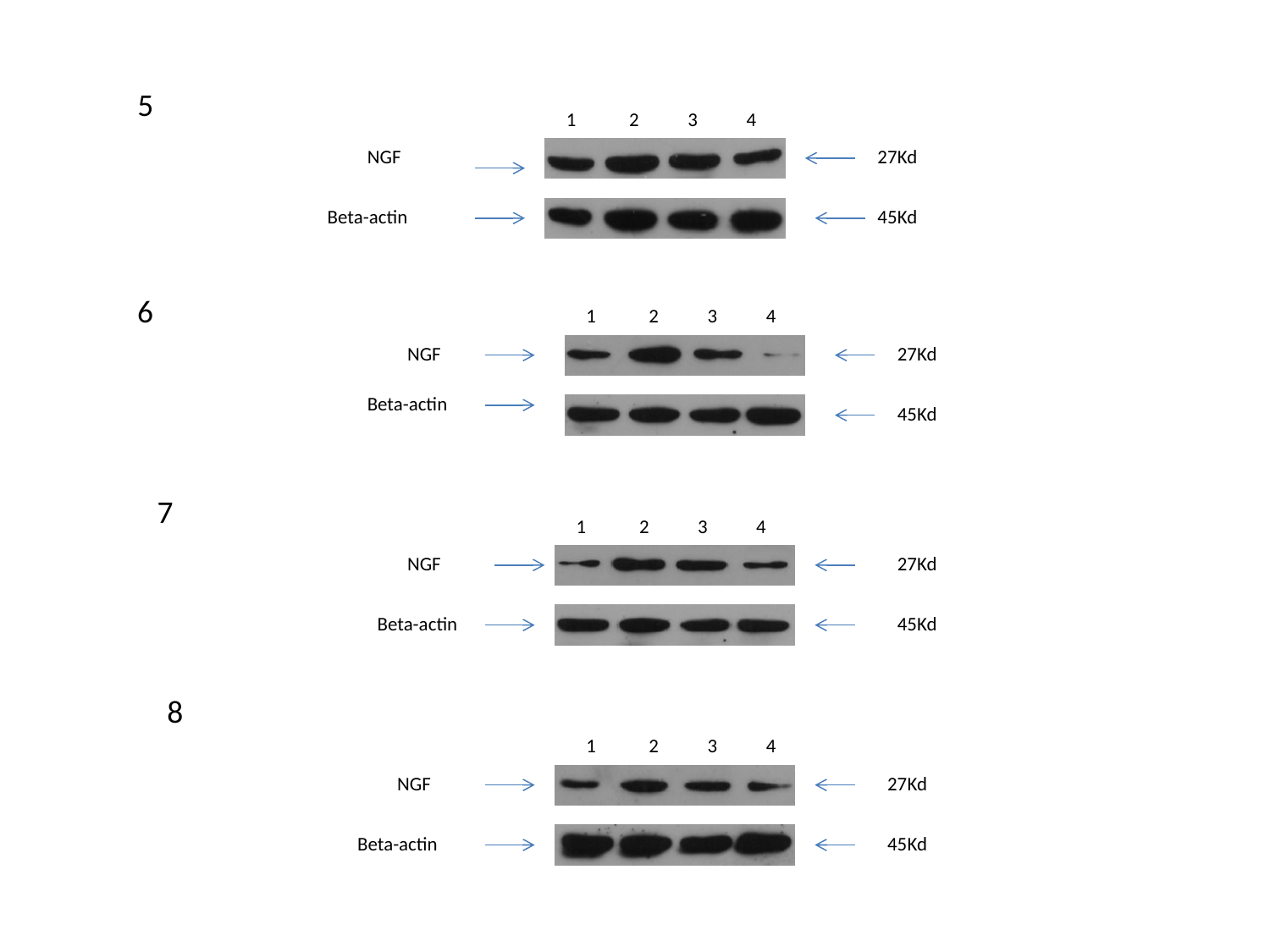

5
 1 2 3 4
NGF
27Kd
Beta-actin
45Kd
6
 1 2 3 4
NGF
27Kd
Beta-actin
45Kd
7
 1 2 3 4
NGF
27Kd
Beta-actin
45Kd
8
 1 2 3 4
NGF
27Kd
Beta-actin
45Kd
