# Supplementary figures and images for: Effects and mechanism of renal denervation on ventricular arrhythmia after acute myocardial infarction in rats
Source: BMC Cardiovasc Disord. 2022 Dec 12;22:544. doi: 10.1186/s12872-022-02980-4 (PMC9743565; doi:10.1186/s12872-022-02980-4)

①

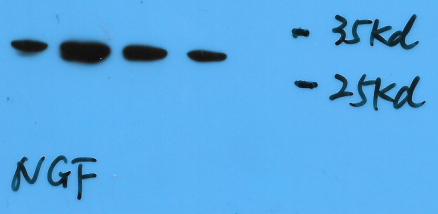

②

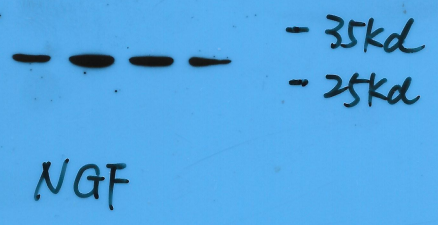

③

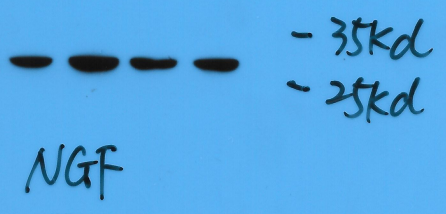

④

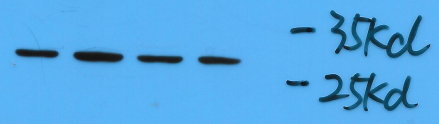

⑤

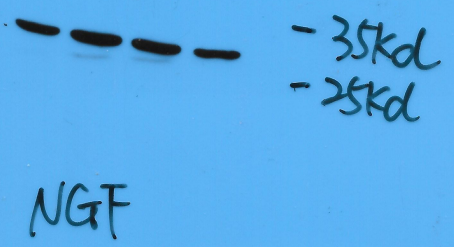

⑥

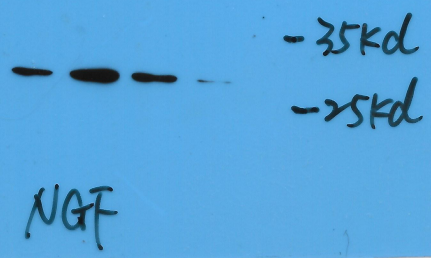

Supplement: Supplementary file 2 — Additional file 2. (Original blot images). [file 12872_2022_2980_MOESM2_ESM.pdf]

⑦

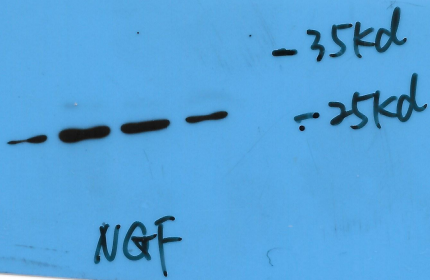

⑧

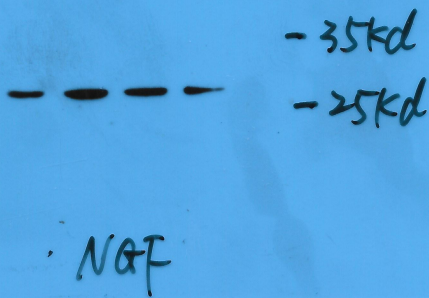

①

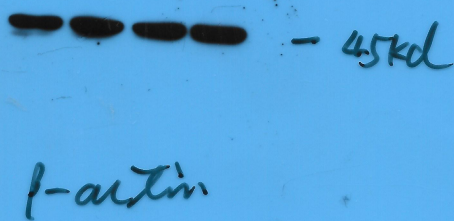

②

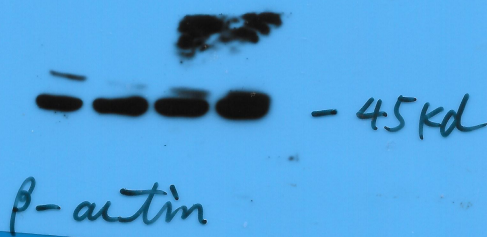

③

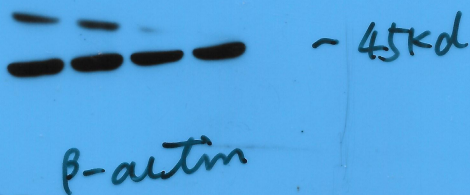

④

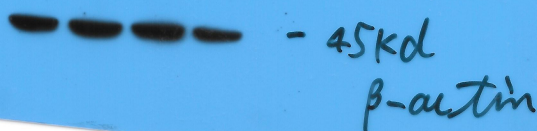

Supplement: Supplementary file 3 — Additional file 3. (Original blot images). [file 12872_2022_2980_MOESM3_ESM.pdf]
