# Supplementary material for: Effects and mechanism of renal denervation on ventricular arrhythmia after acute myocardial infarction in rats
Source: BMC Cardiovasc Disord. 2022 Dec 12;22:544. doi: 10.1186/s12872-022-02980-4 (PMC9743565; doi:10.1186/s12872-022-02980-4)
Supplement: Supplementary file 4 — Additional file 4. (Original blot images). [file 12872_2022_2980_MOESM4_ESM.pdf]

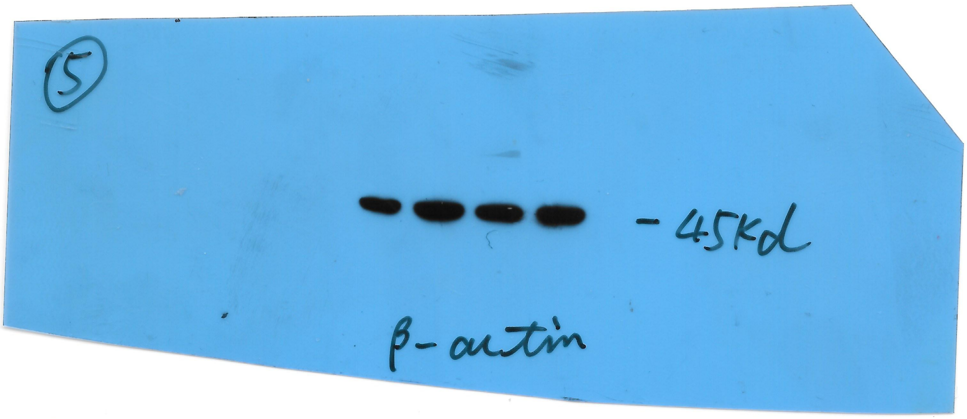
⑤
- 45kd
β-actin

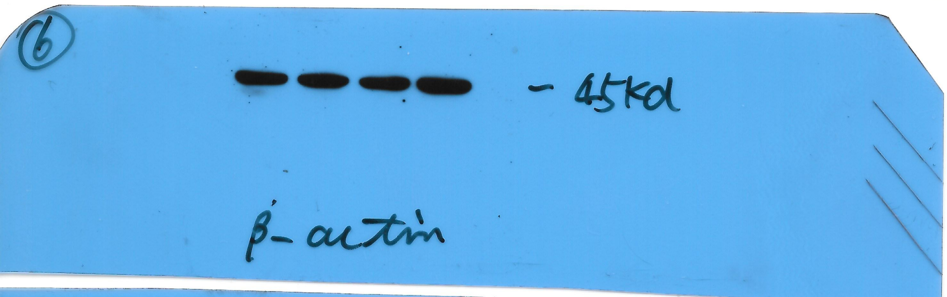
⑥
- 45kd
β-actin

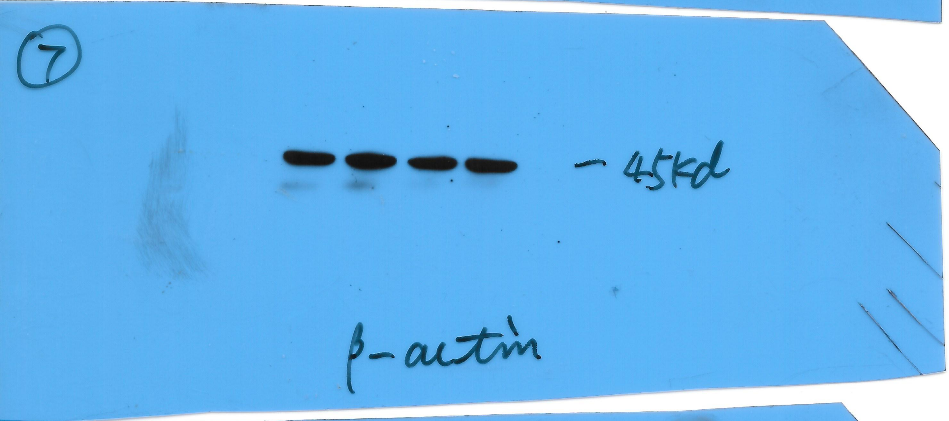
⑦
- 45kd
β-actin

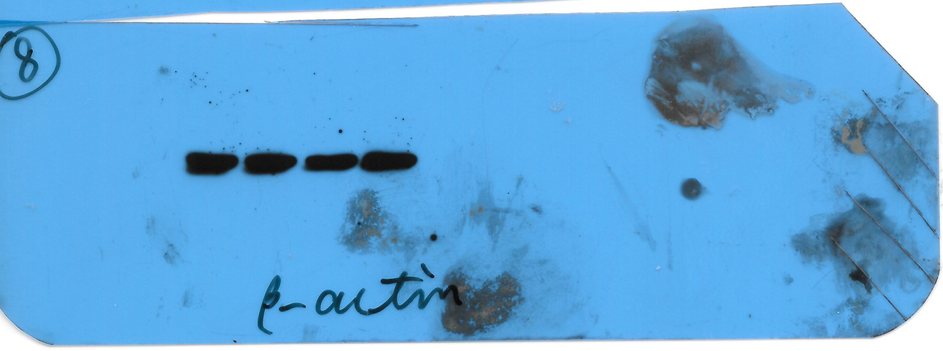
⑧
β-actin
